# Continuum theory of gene expression waves during vertebrate segmentation

**DOI:** 10.1088/1367-2630/17/9/093042

**Published:** 2015-09-24

**Authors:** David J Jörg, Luis G Morelli, Daniele Soroldoni, Andrew C Oates, Frank Jülicher

**Affiliations:** 1 Max Planck Institute for the Physics of Complex Systems, Nöthnitzer Str. 38, D-01187 Dresden, Germany; 2 Departamento de Física, Facultad de Ciencias Exactas y Naturales, Universidad de Buenos Aires, Argentina; 3 IFIBA, CONICET, Pabellón 1, Ciudad Universitaria, 1428 Buenos Aires, Argentina; 4 Francis Crick Institute, Mill Hill Laboratory, The Ridgeway, Mill Hill, London NW7 1AA, UK; 5 Department of Cell and Developmental Biology, University College London, Gower Street, London WC1E 6BT, UK; 6 Max Planck Institute for Cell Biology and Genetics, Pfotenhauerstr. 108, D-01307 Dresden, Germany; julicher@pks.mpg.de

**Keywords:** coupled oscillators, morphogenesis, pattern formation, 05.45.Xt, 82.39.Rt, 87.17.Pq, 87.18.Hf

## Abstract

The segmentation of the vertebrate body plan during embryonic development is a rhythmic and sequential process governed by genetic oscillations. These genetic oscillations give rise to traveling waves of gene expression in the segmenting tissue. Here we present a minimal continuum theory of vertebrate segmentation that captures the key principles governing the dynamic patterns of gene expression including the effects of shortening of the oscillating tissue. We show that our theory can quantitatively account for the key features of segmentation observed in zebrafish, in particular the shape of the wave patterns, the period of segmentation and the segment length as a function of time.

## 1. Introduction

**Figure 1.**
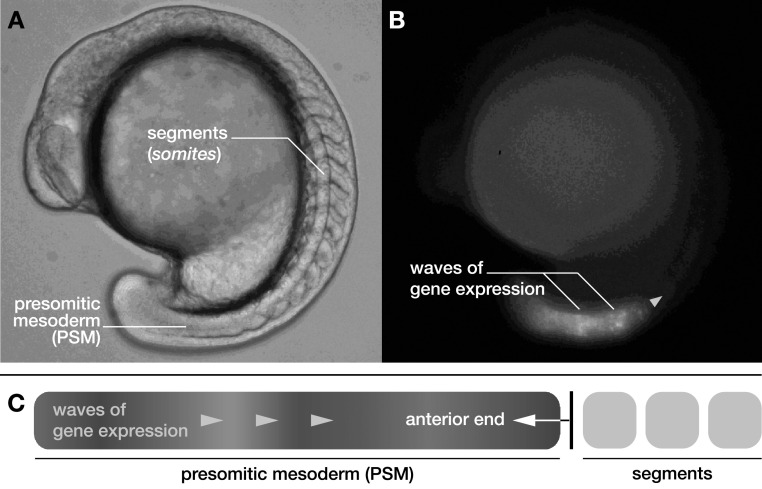
(A) Zebrafish embryo during segmentation of the body axis. (B) The same transgenic embryo as in (A) in the Her1::YFP fluorescence channel highlighting regions of oscillatory gene expression [4]. The green arrow indicates the propagation direction of the waves. (C) A Doppler effect occurs as the anterior end moves into the waves due to PSM shortening.

In all vertebrate animals, the segmentation of the body plan proceeds during embryonic development in a process termed *somitogenesis* [1]. During somitogenesis, the elongating body axis segments rhythmically and sequentially into *somites*, the precursors of vertebrae and ribs. Failure of proper segmentation, caused for instance by mutations, can give rise to birth defects such as congenital scoliosis [2]. Somites are formed in characteristic time intervals from an unsegmented progenitor tissue, the *presomitic mesoderm* (PSM) (figure 1(A)). The temporal regularity with which somites form has provoked the idea that a biological clock comprised of cellular oscillators coordinates the temporal progress of segmentation in the PSM. The so-called ‘clock-and-wavefront’ mechanism suggests that a wavefront at the anterior end of the PSM reads out the state of this clock and triggers the formation of a new segment upon each completed clock cycle [3]. Indeed, patterns of oscillating gene expression have been found in the PSM of various vertebrates such as zebrafish, chick, mouse, frog, and snake [1]. These patterns resemble traveling waves sweeping through the PSM and occur as a result of coordinated cellular oscillations in the concentration of gene products (figure 1(B)). Genetic oscillations are proposed to occur autonomously in single cells as a result of delayed autorepression of specific genes [5, 6]. Cellular oscillators mutually couple through Delta–Notch signaling between neighboring cells, which tends to locally synchronize their oscillatory dynamics [7–11]. Local synchronization due to coupling is important to maintain coherent wave patterns by preventing the cellular oscillators from drifting out of phase due to noise in gene expression [12–14]. The emergence of traveling waves at the tissue level has been linked to a gradual slowdown of genetic oscillations in the PSM along the body axis [1, 13, 15, 16]. This gradual slowdown corresponds to a spatial profile of intrinsic frequencies of the cellular oscillators.

During segmentation, the waves of gene expression emerge at the posterior of the PSM and travel towards its anterior end, where the new segments are formed (figure 1(B)). Segment formation occurs upon arrival of a wave at the anterior end of the PSM. This corresponds to the formation of one segment with each completed oscillation cycle at the anterior end [4]. Segmentation is a highly dynamic process: in parallel with segment formation, the body axis elongates while at the same time PSM changes its length as cells leave the PSM at the anterior end to form somites [4, 16]. A shortening of the PSM, as observed in Zebra fish moves relative to the waves giving rise to a Doppler effect (figure 1(C)) [4]. The motion of the anterior end relative to the posterior tip leads to an increase of the frequency of oscillations seen by an observer at the anterior end. Since the oscillation frequency at the anterior end specifies the rate of segmentation, this Doppler effect contributes to a decrease of the period of morphological segment formation. In addition to the Doppler effect, the wavelength of the pattern dynamically changes over time. This leads to a modulation of the local frequency and contributes to an increase of the period of segmentation. Together, both effects combine to determine the timing of segment formation. Hence, in addition to the time scale of genetic oscillations, the rate of segment formation is regulated by the time scale set by tissue shortening and the wavelength of the wave pattern. These observations highlight the need to capture the effects of tissue deformation in theories of vertebrate segmentation.

In this paper, we present a minimal continuum theory of vertebrate segmentation based on coupled phase oscillators in a dynamic medium that takes into account local growth and shortening of the oscillating tissue during the segmentation process. In section 2, we introduce our continuum theory of vertebrate segmentation and the key observables that can be obtained from the theory. In section 3, we illustrate the basic mechanism of pattern formation with oscillators using a simplified scenario with constant length of the oscillating tissue. In section 4, we apply our theory to quantitatively describe segmentation in developing zebrafish embryo, taking into account tissue shortening. In section 5, we discuss the factors that regulate the period of segmentation and show how a Doppler effect and a dynamic wavelength effect emerge from the interplay of tissue shortening and changing wave patterns. In section 6, we discuss our findings and give an outlook for further research.

## 2. Continuum theory of coupled oscillators in a dynamic medium

**Figure 2.**
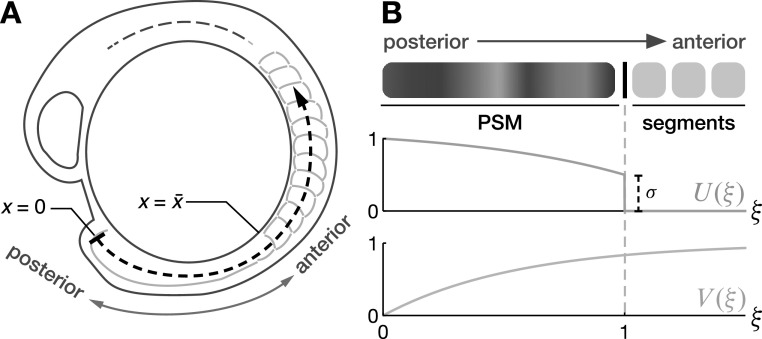
(A) Curved coordinate axis for the zebrafish embryo. The point *x* = 0 marks the posterior tip of the PSM and sets the reference frame. (B) Shape of the profiles *U* and *V*, equations (4) and (5).

Here we introduce a theory that aims to describe the wave patterns in the PSM and the dynamic features of segmentation that result from these wave patterns. The wave patterns and the timing of segmentation have previously been quantified in transgenic zebrafish embryos, in which oscillating genes have been tagged with a fluorescent marker protein [4]. Waves can be traced by introducing a one-dimensional coordinate *x* along the curved embryonic body axis and measuring the fluorescent intensity level along this axis over time (figures 1(B) and 2(A)). Since these wave patterns are a tissue-level phenomenon and phase differences between neighboring cellular oscillators are typically small, we here choose a coarse-grained continuum description of the oscillatory medium. We describe the local state of oscillation by a phase field $\phi (x,t).$ Our theory combines three key ingredients involved in pattern formation during vertebrate segmentation: (i) autonomous oscillators with a spatial profile $\omega (x)$ of intrinsic frequencies [13, 15], (ii) local oscillator coupling with strength *ϵ* [10, 13], and (iii) a cell velocity field *v*(*x*) capturing deformation and elongation of the segmenting body axis [17, 18]. The dynamic equation for the phase field *ϕ* is given by [13]

1 \begin{eqnarray*}\displaystyle \frac{\partial \phi }{\partial t}+v\displaystyle \frac{\partial \phi }{\partial x}=\omega +\displaystyle \frac{\varepsilon }{2}\displaystyle \frac{{\partial }^{2}\phi }{\partial {x}^{2}}.\end{eqnarray*}

The intrinsic frequency of the oscillators is described by a position-dependent frequency profile $\omega (x).$ Motion of the cellular oscillators is described by an advective term where *v* is the cell velocity. In previous work, we have considered a constant velocity *v*. Local oscillator coupling with strength *ϵ* is described by a term that tends to even out local phase differences and thus describes the oscillators’ tendency to locally synchronize [19]. We impose open boundary conditions, $(\partial \phi /\partial x){| }_{x=0}=0,$ which corresponds to the situation where there are no oscillators beyond the posterior tip.

In order to describe a shortening PSM, we consider the simple case where the frequency and the velocity profile are rescaled with tissue length

2 \begin{eqnarray*}\omega ={\omega }_{0}U(x/\bar{x}(t)),\end{eqnarray*}

3 \begin{eqnarray*}v={v}_{0}V(x/\bar{x}(t)),\end{eqnarray*}

where *U* and *V* are spatial profiles that are adjusted to the variant length $\bar{x}(t)$ of the PSM, ${\omega }_{0}$ is the maximum frequency at the posterior tip *x* = 0, and *v*_0_ is a typical velocity.

Phase waves travel in an anterior direction if the frequency profile attains its maximum frequency at the posterior tip *x* = 0 and decays in an anterior direction [13, 15]. For simplicity, we consider that oscillations have ceased beyond the wavefront and therefore choose the following frequency profile

4 \begin{eqnarray*}U(\xi )=\left\{\begin{array}{ll}\sigma +(1-\sigma )\displaystyle \frac{1-{{\rm{e}}}^{k(\xi -1)}}{1-{{\rm{e}}}^{-k}} &amp; \xi \leqslant 1\\ 0 &amp; \xi \gt 1,\end{array}\right.\end{eqnarray*}

see figure 2(B), where $\xi =x/\bar{x}$ denotes a non-dimensional position coordinate and *k*^−1^ is a characteristic (non-dimensional) length scale of the profile. The function *U* has the boundary values $U(0)=1$ and $U(1)=\sigma $ (figure 2(B)).

The velocity field in the segmented region can be estimated from experiments by tracking the velocity of segment boundaries, see appendix A. Choosing the boundary condition $v(0)=0,$ a simple choice for the velocity profile consistent with the quantified data is

5 \begin{eqnarray*}V(\xi )=1-{{\rm{e}}}^{-q\xi },\end{eqnarray*}

see figure 4(B). The velocity gradient *v* corresponds to local growth rate with a profile $\partial v/\partial x=({{qv}}_{0}/\bar{x}){{\rm{e}}}^{-{qx}/\bar{x}}$ that takes its maximum value at the posterior tip *x* = 0 and decays over the characteristic length scale $\bar{x}/q.$ The choice of the functional forms for *U* and *V* are motivated by experimental observations as they give rise to the type of wave patterns observed in experiments with waves moving in anterior direction and slowing down as they approach the anterior end, see section 3.

**Figure 3.**
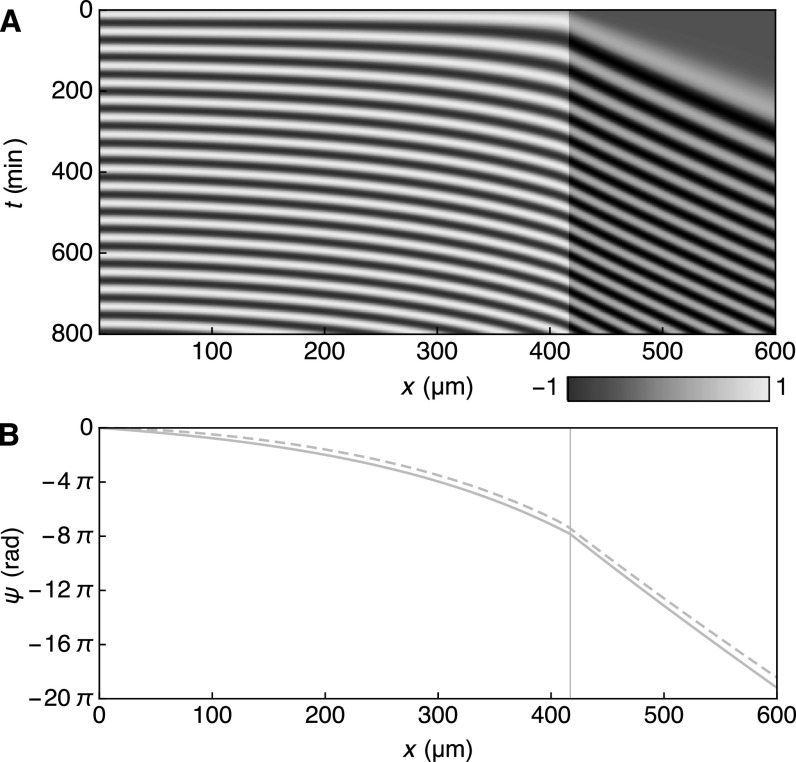
(A) Kymograph of a numerical solution to equation (1) with constant PSM length $x={\bar{x}}_{0}.$ The density plot displays $\mathrm{sin}\phi (x,t)$ (see color legend). The PSM region $x\lt {\bar{x}}_{0}$ is displayed in blue, the segmented part $x\gt {\bar{x}}_{0}$ in gray. (B) Stationary phase profile *ψ*, defined by equation (9), as obtained from numerical solutions of equation (1) (solid) and the approximation equation (11) (dashed), which neglects the effects of coupling. Parameters are specified in table 1.

**Figure 4.**
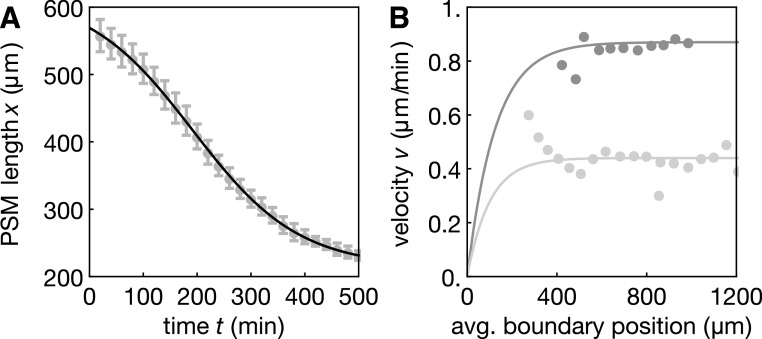
(A) Time evolution of the average PSM length $\bar{x},$ from experimental data (dots) and the analytical fit function equation (14) (black curve) with parameters given in table 1. Dots show averages over 18 embroys, error bars indicate standard deviation. Data from [4]. (B) Velocities of segment boundaries as a function of the average boundary position (appendix A). Dots show experimental data, curves show functions *v*, equation (3). Dark green: velocities during formation of segments 10–17, *v*_0_ = 0.87 *μ*m min^−1^, $q/\bar{x}=0.008\;\mu {\rm{m}}$ (dark) and *v*_0_ = 0.44 *μ*m min^−1^, $q/\bar{x}=0.01\;\mu {\rm{m}},$ segments 18–22 (bright).

The number of waves that simultaneously sweep through the PSM is a key observable that can be measured in experiments [1]. In terms of the phase field *ϕ*, the number of waves *K*(*t*) is given by

6 \begin{eqnarray*}K(t)=\displaystyle \frac{\phi (0,t)-\phi (\bar{x}(t),t)}{2\pi }.\end{eqnarray*}

Hence, $2\pi K$ is the total phase difference between the posterior tip *x* = 0 and the anterior end $x=\bar{x}$ of the PSM. A new segment is formed after each completed oscillation cycle at the anterior end $x=\bar{x}$ [4]. Accordingly, the number of formed segments at time *t* is given by

7 \begin{eqnarray*}N(t)=\displaystyle \frac{\phi (\bar{x}(t),t)}{2\pi }\end{eqnarray*}

and the rate of segment formation is ${\rm{d}}N/{\rm{d}}t.$ The length *S* of the formed segments at the time *t* of their formation is given by the wavelength of the pattern at the anterior end, and obeys $| \phi (\bar{x},t)-\phi (\bar{x}+S(t),t)| =2\pi .$ In the case where $\partial \phi /\partial x$ does not vary strongly over the length *S*, the segment length can be approximated as

8 \begin{eqnarray*}S(t)\simeq \displaystyle \frac{2\pi }{\left|\displaystyle \frac{\partial \phi }{\partial x}(\bar{x}(t),t)\right|}.\end{eqnarray*}

## 3. Time-periodic patterns

We first discuss time-periodic patterns to illustrate how the properties of the wave pattern depend on the parameters of our theory. Such patterns occur for constant PSM length, $\bar{x}(t)={\bar{x}}_{0}.$ Figure 3(A) shows a kymograph of a time-periodic solution to equation (1). Starting with $\phi (x,t=0)=0,$ the system attains a time-periodic state after transient dynamics. This time-periodic state can be expressed in the form [13, 19]

9 \begin{eqnarray*}\phi (x,t)={\rm{\Omega }}t+\psi (x),\end{eqnarray*}

where Ω is the collective frequency and the spatiotemporal pattern $\mathrm{sin}\phi (x,t)$ is fully characterized by the time-independent phase profile $\psi (x).$ The rate of segment formation ${\rm{d}}N/{\rm{d}}t,$ defined through equation (7), is given by ${\rm{d}}N/{\rm{d}}t={\rm{\Omega }}/2\pi $ and hence given by the collective frequency. Using the time-periodic ansatz equation (9) in equation (1), the phase profile *ψ* obeys the ordinary differential equation

10 \begin{eqnarray*}{\rm{\Omega }}+v{\psi }^{\prime }=\omega +\displaystyle \frac{\varepsilon }{2}{\psi }^{\prime\prime }\end{eqnarray*}

with boundary condition ${\psi }^{\prime }(0)=0.$ It is instructive to consider the case of weak coupling, in which the coupling term provides only a minor correction to the collective frequency and the phase profile (figure 3(B)). Neglecting $(\varepsilon /2){\psi }^{\prime\prime }$ in equation (10), we find the collective frequency ${\rm{\Omega }}\simeq {\omega }_{0},$ the maximum of the frequency profile at the posterior tip. The phase profile *ψ* can then be approximated as

11 \begin{eqnarray*}\psi (x)\simeq {\displaystyle \int }_{0}^{x}\displaystyle \frac{\omega (x^{\prime} )-{\rm{\Omega }}}{v(x^{\prime} )}\;{\rm{d}}x^{\prime} .\end{eqnarray*}

Figure 3(B) shows the approximation equation (11) together with the phase profile obtained from a numerical solution of equation (1) including the effects of coupling. The number of waves that simultaneously sweep through the PSM is given by $K=| \psi ({\bar{x}}_{0})| /2\pi .$ The length *S* of formed segments is constant and given by equation (8) as

12 \begin{eqnarray*}S=\displaystyle \frac{2\pi }{| {\psi }^{\prime }({\bar{x}}_{0})| }\simeq {v}_{0}T,\end{eqnarray*}

where we have approximated $v({\bar{x}}_{0})\simeq {v}_{0}$ and defined the collective period $T=2\pi /{\rm{\Omega }}.$ This relationship is well-known from the clock-and-wavefront model [3, 13]. Note that in the case of a velocity profile it only holds approximately and only for time-periodic solutions. The phase velocity $\tilde{v}={\rm{d}}{x}_{*}/{\rm{d}}t$ of the waves can be obtained as the velocity of a point x_*_ with constant phase, $\phi ({x}_{*}(t),t)={\phi }_{*}$ [20]. Differentiating this relation with respect to time yields the phase velocity $\tilde{v}=-(\partial \phi /\partial t)/(\partial \phi /\partial x){| }_{x={x}_{*}(t)},$ which exists at any position *x*. Using equations (9) and (11), we obtain

13 \begin{eqnarray*}\tilde{v}(x)\simeq \displaystyle \frac{v(x)}{1-\omega (x)/{\omega }_{0}}.\end{eqnarray*}

The phase velocity $\tilde{v}(x)$ is always positive and larger than *v*(*x*) because $0\lt \omega (x)\leqslant {\omega }_{0}.$ This implies that the waves move in anterior direction and faster than the underlying medium moves away from the tip.

## 4. Dynamic patterns in a shortening tissue

We now consider the more realistic situation where the oscillating tissue changes its length as is the case for the PSM in developing vertebrate embryos. Here we focus on the spatiotemporal pattern of the oscillating gene Her1. The patterns of this gene product can be observed *in vivo* by a fluorescent label that is introduced in the transgenic zebrafish line *Looping* [4]. In zebrafish, the PSM substantially shortens during segmentation [4]. The time dependence of the PSM length $\bar{x}(t)$ can be well captured by the function [4]

14 \begin{eqnarray*}\bar{x}(t)={x}_{0}+{x}_{1}\mathrm{tanh}\eta (t-\bar{t}).\end{eqnarray*}

Figure 4(A) shows this function with parameters given in table 1 together with experimental data points from [4]. Here, *t* = 0 corresponds to the formation time of the 7th segment. We now discuss our model taking into account this time dependence of the PSM length.

**Table 1.** Parameters used for the phase model equation (1) to describe segmentation of the transgenic zebrafish line *Looping* at 23.5 °C.

Param.	Value	Description	Source
${\omega }_{0}$	$0.15\ \mathrm{rad}\;{\mathrm{min}}^{-1}$	Maximum frequency	[4]
*v*_0_	$0.87\ \mu {\rm{m}}\;{\mathrm{min}}^{-1}$	Maximum velocity	Quantified (appendix A)
*ϵ*	$7\ \mu {{\rm{m}}}^{2}\;{\mathrm{min}}^{-1}$	Coupling strength	[14, 19]
*k*	2.07	Frequency profile shape parameter	Fit (appendix B)
*σ*	0.34	Frequency profile shape parameter	Fit (appendix B)
*q*	1.80	Velocity profile shape parameter	Fit (appendix B)
*t*_0_	$-256\ \mathrm{min}$	Initial time	Fit (appendix B)
*x*_0_	$417\ \mu {\rm{m}}$	Parameters of the time-dep.	[4]
*x*_1_	$202\ \mu {\rm{m}}$	PSM length equation (14)
*η*	$-5.09\times {10}^{-3}\ {\mathrm{min}}^{-1}$	
$\bar{t}$	$192\ \mathrm{min}$		

**Figure 5.**
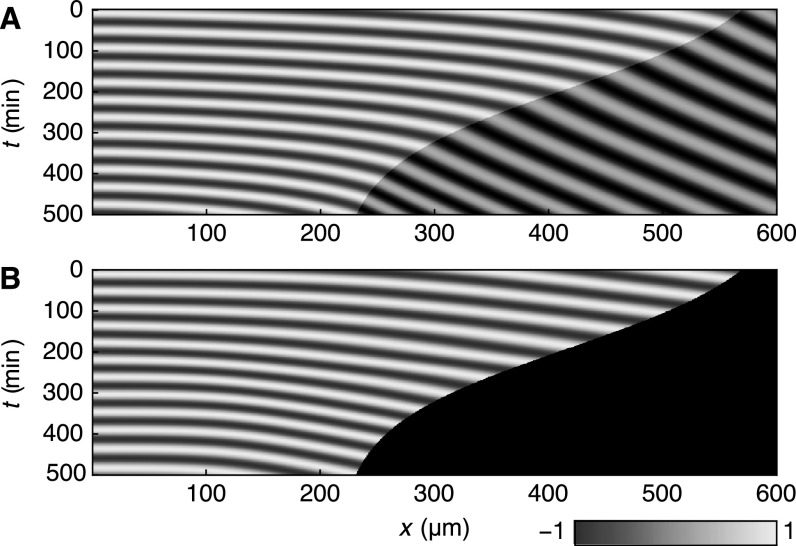
(A) Kymograph of a numerical solution to equation (1) with time-dependent PSM length $\bar{x}(t),$ equation (14). Color code as in figure 3. Parameters are specified in table 1. (B) Kymograph of the experimentally obtained average phase patterns in transgenic zebrafish embryos from [4].

Figure 5(A) shows a kymograph of a numerical solution to equations (1)–(3) using equation (14). The experimentally obtained phase profile from [4] is shown in figure 5(B) for comparison. Comparison of figures 5(A) and (B) show that the theoretical and experimental wave patterns qualitatively agree. Parameters were chosen such that the theory captures the features of the experimentally obtained wave patterns: the velocity *v*_0_ in the segmented region was obtained from quantification of segment boundary positions as a function of time (figure 4(B)), see appendix A. The remaining parameters were obtained from fits of the theoretical phase profile to the experimental wave pattern shown in figure 5(B) (for fit procedures see appendix B). An alternative way to display the wave pattern is to introduce the time-dependent phase profile

**Figure 6.**
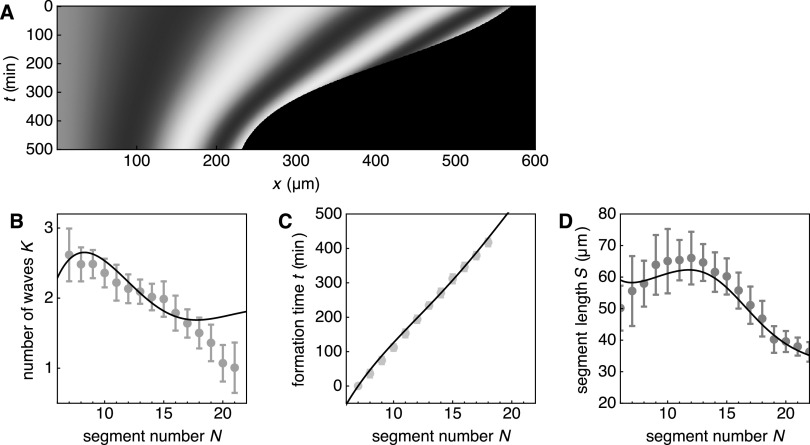
(A) Kymograph of the phase profile $\psi (x,t),$ equation (15) of the same numerical solution of the theory as shown in figure 5(A). (B) Number of waves *K*, equation (6), as a function of the segment number *N* from experiments (blue dots) and theory (black curve). (C) Formation time *t* of segment number *N* from experiments (green dots) and from theory (black curve), equation (7). Experimental data points are averages over 18 embryos. (D) Segment length *S* at time of segment formation from experiments (red dots) and from theory (black curve), equation (8). Experimental data points are averages over ten embryos. Error bars in both plots indicate the standard deviation.

15 \begin{eqnarray*}\psi (x,t)=\phi (x,t)-\phi (0,t),\end{eqnarray*}

see figure 6(A). Note that for time-periodic solutions this becomes the time-independent phase profile defined in equation (9). Figure 6(A) reveals that the wavelength of pattern decreases over time as wave peaks are moving closer together. Furthermore, it can be seen that the number of waves in the PSM decreases over time as the anterior end cuts off one wave peak while the PSM is shortening. The fact that the number of waves in the PSM changes over time shows that the phase profile does not simply scale with the PSM length. Figure 6(B) shows the number of waves as a function of the number of formed segments both from numerical solutions of the phase model and from experiments as presented in [4]. The number of waves substantially decreases during segmentation, which is captured well by the theory (figure 6(B)). The discrepancy between the solid line in figure 6(B) and the experimental data for segments $N\geqslant 18$ suggests that the scaling frequency and velocity profiles, equations (2) and (3), are too simple to capture the wave patterns at late times.

Our theory can also quantitatively account for the features of morphological segment formation. Figures 6(C) and (D) show a comparison of our theory to experiments for the formation time and segment length as a function of the segment number *N*, respectively (for details see appendix A). The segment length *S* shows a non-monotonic behavior with largest segments being formed around the 12-segment mark, a behavior also found in wildtype zebrafish [21]. This demonstrates that our theory can quantitatively account for the dynamic features of vertebrate segmentation.

## 5. Doppler and dynamic wavelength effect

### 5.1. Period of segmentation

A fundamental feature of segmentation is that segments are formed rhythmically and sequentially. Which factors determine the period of morphological segment formation? From the definition equation (7) of the segment number *N*, it follows that the rate of segmentation is given by the local frequency at the moving anterior end, ${\rm{d}}N/{\rm{d}}t={{\rm{\Omega }}}_{{\rm{A}}}/2\pi ,$ where

16 \begin{eqnarray*}{{\rm{\Omega }}}_{{\rm{A}}}=\displaystyle \frac{{\rm{d}}}{{\rm{d}}t}\phi (\bar{x}(t),t).\end{eqnarray*}

Hence, the rate of segment formation is generally time-dependent. We now show how the wave pattern influences Ω_A_. To this end, we decompose Ω_A_ into different contributions [4]

17 \begin{eqnarray*}{{\rm{\Omega }}}_{{\rm{A}}}={{\rm{\Omega }}}_{{\rm{P}}}+{{\rm{\Omega }}}_{{\rm{D}}}+{{\rm{\Omega }}}_{{\rm{W}}},\end{eqnarray*}

where Ω_P_ is the posterior frequency, Ω_D_ is a Doppler contribution and Ω_W_ is a ‘dynamic wavelength’ contribution. These frequencies are defined by

18 \begin{eqnarray*}{{\rm{\Omega }}}_{{\rm{P}}} &amp; = &amp; \displaystyle \frac{\partial \phi }{\partial t}(0,t),\\ {{\rm{\Omega }}}_{{\rm{D}}} &amp; = &amp; \displaystyle \frac{{\rm{d}}\bar{x}}{{\rm{d}}t}\displaystyle \frac{\partial \psi }{\partial x}(\bar{x}(t),t),\\ {{\rm{\Omega }}}_{{\rm{W}}} &amp; = &amp; \displaystyle \frac{\partial \psi }{\partial t}(\bar{x}(t),t),\end{eqnarray*}

where the phase profile *ψ* is defined in equation (15). The contribution Ω_P_ is the local frequency at the posterior tip of the tissue at *x* = 0. The contribution Ω_D_ results from a Doppler effect where ${\rm{d}}\bar{x}/{\rm{d}}t$ is the speed of the moving observer (the anterior end) traveling into a wave with wavelength $2\pi {(\partial \psi /\partial x)}^{-1}.$ The contribution Ω_W_ is caused by the change of the phase profile *ψ* over time, which corresponds to a dynamic change of the wavelength.

Using our theory, we can derive an explicit relation between Ω_A_ and Ω_P_ for the simple case of linear shortening of the PSM, ${\rm{d}}\bar{x}/{\rm{d}}t=-\bar{v},$ see appendix C. We find

19 \begin{eqnarray*}{{\rm{\Omega }}}_{{\rm{A}}}\simeq \left(1+\displaystyle \frac{\bar{v}}{{v}_{0}}\right)(1-{\rm{\Delta }}){{\rm{\Omega }}}_{{\rm{P}}},\end{eqnarray*}

where

20 \begin{eqnarray*}{\rm{\Delta }}={\displaystyle \int }_{0}^{1}\displaystyle \frac{\bar{v}/{v}_{0}}{{(1+\bar{v}\xi /{v}_{0})}^{2}}\displaystyle \frac{\omega (\xi )}{{\omega }_{0}}\;{\rm{d}}\xi .\end{eqnarray*}

In equation (19), the factor $1+\bar{v}/{v}_{0}$ describes the Doppler effect with the speed $\bar{v}$ of the moving observer (the anterior end) and the cell velocity *v*_0_. The factor $1-{\rm{\Delta }}$ describes the effects caused by changing phase profile due to the shortening of the frequency profile with the PSM length. Hence, this term describes the dynamic wavelength effect. Because ${\rm{\Delta }}\gt 0,$ this factor opposes the Doppler effect.

**Figure 7.**
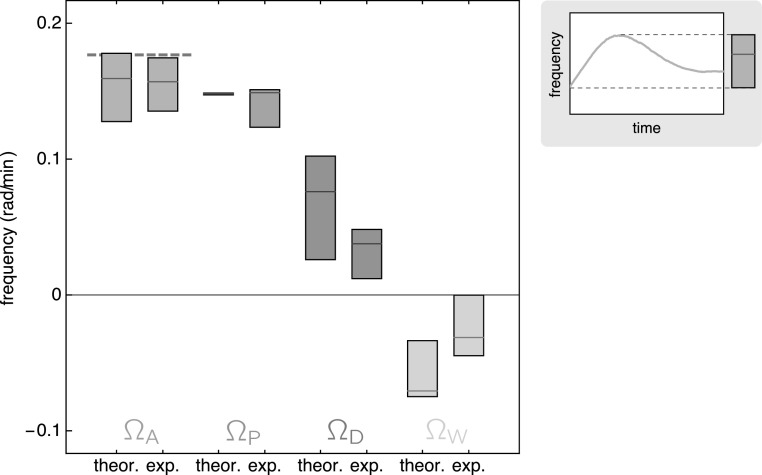
Distribution of frequency values over the entire range of time (500 min): anterior frequency Ω_A_ (blue), equation (17), and its contributions, equation (18): the posterior frequency Ω_P_ (purple), the Doppler contribution Ω_D_ (red), and the dynamic wavelength contribution Ω_W_ (green), for the theoretical and experimental systems displayed in figure 5. Boxes show the total range of values over time with the horizontal line indicating the median (see schematic outset plot). The dashed blue line indicates the approximation equation (19) in the time interval between 150 and 300 min where PSM shortening is approximately linear with $\bar{v}=1.03\ \mu {\rm{m}}\;{\mathrm{min}}^{-1},$ see figure 4(A). Experimental data from [4].

Figure 7 displays theoretical and experimental results for the anterior frequency Ω_A_ and the contributions Ω_P_, Ω_D_, and Ω_W_, together with the approximation equation (19) for Ω_A_. The Doppler effect yields a positive contribution (${{\rm{\Omega }}}_{{\rm{D}}}\gt 0$), the dynamic wavelength yields a negative contribution (${{\rm{\Omega }}}_{{\rm{W}}}\lt 0$) with the Doppler effect having larger magnitude, consistent with experiments [4]. The average anterior frequency Ω_A_ is thus larger than the posterior frequency Ω_P_.

The Doppler effect and the dynamic wavelength effect can be discussed in the context of classical wave physics.

### 5.2. Doppler effect

Consider a wave equation in one-dimension

21 \begin{eqnarray*}\displaystyle \frac{{\partial }^{2}u}{\partial {t}^{2}}-{c}^{2}\displaystyle \frac{{\partial }^{2}u}{\partial {x}^{2}}=0,\end{eqnarray*}

where $u(x,t)$ is the amplitude of the wave and *c* is the wave propagation speed. We consider a wave-emitting source with frequency *ω* and amplitude *u*_0_ at *x* = 0 through the boundary condition

22 \begin{eqnarray*}u(0,t)={u}_{0}\mathrm{sin}\omega t.\end{eqnarray*}

Furthermore, we impose the zero initial conditions

23 \begin{eqnarray*}u(x,0)=0.\end{eqnarray*}

A simple solution to equation (21) satisfying the boundary and initial conditions (22) and (23) is

24 \begin{eqnarray*}u(x,t)={u}_{0}\mathrm{sin}(\omega t-2\pi x/\lambda ),\end{eqnarray*}

a plane wave with wavelength $\lambda =2\pi c/\omega .$ The phase pattern of this wave is $\phi (x,t)=\omega t-{qx}.$ An observer with position $\bar{x}(t)$ moving with constant velocity ${\rm{d}}\bar{x}/{\rm{d}}t=-\bar{v}$ observes the frequency ${\rm{\Omega }}={\rm{d}}\bar{\phi }/{\rm{d}}t$ with $\bar{\phi }(t)=\phi (\bar{x}(t),t).$ Using

25 \begin{eqnarray*}\displaystyle \frac{{\rm{d}}\bar{\phi }}{{\rm{d}}t}={\left.\left(\displaystyle \frac{\partial \phi }{\partial t}+\displaystyle \frac{\partial \phi }{\partial x}\displaystyle \frac{{\rm{d}}\bar{x}}{{\rm{d}}t}\right)\right|}_{x=\bar{x}(t)},\end{eqnarray*}

we have

26 \begin{eqnarray*}{\rm{\Omega }}=\omega +{{\rm{\Omega }}}_{{\rm{D}}},\end{eqnarray*}

where

**Figure 8.**
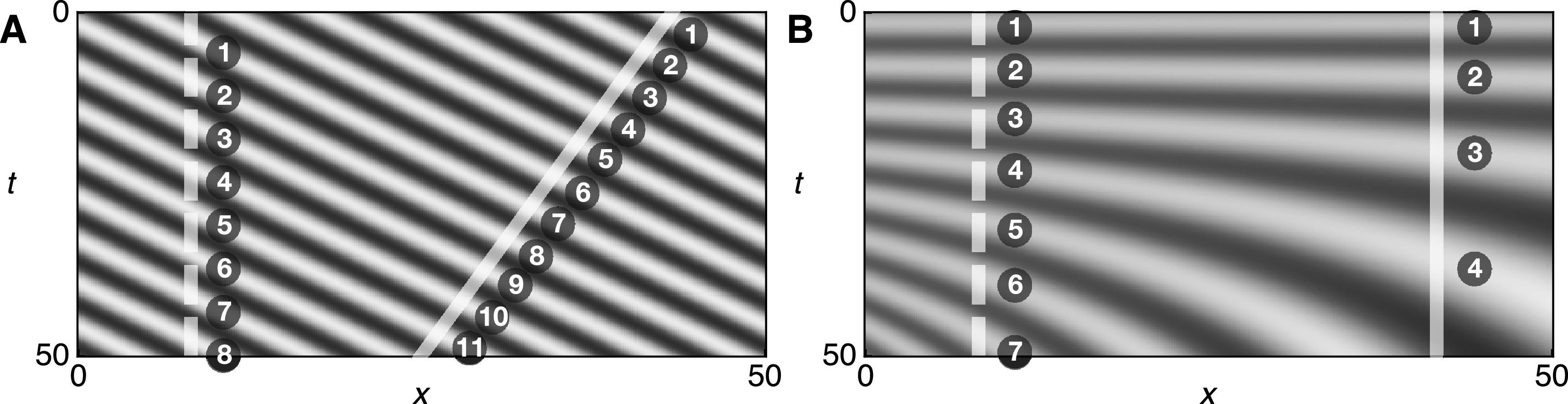
(A) Kymograph of the plane wave $u(x,t),$ equation (24). The semi-transparent white lines indicate an observer at rest (dashed) and an observer in motion (solid) having constant velocity ${\rm{d}}\bar{x}/{\rm{d}}t=-\bar{v}.$ The numbers indicate the wave peaks that the respective observer crosses. (B) Kymograph of the wave $u(x,t)$ in a medium with time-dependent refractive index, equation (30). The semi-transparent white lines indicate two observers at rest with different positions. Parameters are $\omega =1,$ *c* = 1, ${u}_{0}=1,$ $r=5\times {10}^{-4}.$ The color code is the same as in figure 5. In panel (B), the color code has been rescaled to the minimum and maximum values of *u*.

27 \begin{eqnarray*}{{\rm{\Omega }}}_{{\rm{D}}}=\displaystyle \frac{{\rm{d}}\bar{x}}{{\rm{d}}t}\displaystyle \frac{\partial \phi }{\partial x}(\bar{x}(t),t),\end{eqnarray*}

which corresponds to Ω_D_ in equation (18). Note that ${\rm{\Omega }}=(1+\bar{v}/c)\omega ,$ which is the usual expression for the Doppler effect of a moving observer [22]. The wave pattern described by equation (24) is shown as a kymograph in figure 8(A). This pattern can be used to illustrate the Doppler effect by considering an observer at rest (dashed white line) compared to an observer moving towards the source (solid white line). The moving observer crosses more wave peaks as compared to the observer at rest during the same time interval and hence observes a higher frequency.

### 5.3. Dynamic wavelength effect

A dynamic wavelength effect, i.e., a time-dependent change of the wavelength at a fixed point in space, can occur if waves propagate in a medium with a time-dependent index of refraction $n(t).$ In this case, the dynamic equation for the waves is given by

28 \begin{eqnarray*}\displaystyle \frac{{\partial }^{2}u}{\partial {t}^{2}}-\displaystyle \frac{{c}^{2}}{n{(t)}^{2}}\displaystyle \frac{{\partial }^{2}u}{\partial {x}^{2}}=0,\end{eqnarray*}

To illustrate how the dynamic wavelength effect emerges, we here consider for simplicity

29 \begin{eqnarray*}n(t)={{rt}}^{2},\end{eqnarray*}

For this case, a solution to equation (28) with the boundary and initial conditions (22) and (23) is given by

30 \begin{eqnarray*}u(x,t)={u}_{0}\left(1+\displaystyle \frac{r}{c}{xt}\right)\mathrm{sin}\left(\displaystyle \frac{\omega t}{1+\displaystyle \frac{r}{c}{xt}}\right)\end{eqnarray*}

In this case, the phase profile is given by $\phi (x,t)=\omega t/(1+{rxt}/c).$ The prefactor ${u}_{0}(1+{rxt}/c)$ describes a position and time-dependent wave amplitude. Equation (30) describes waves which propagates with a phase velocity $\tilde{v}=-(\partial \phi /\partial t)/(\partial \phi /\partial x)=c/n(t).$ The local wavelength $\lambda =2\pi /| \partial \phi /\partial x| $ at position *x* and time *t* is given by

31 \begin{eqnarray*}\lambda (x,t)=\displaystyle \frac{2\pi }{{rc}\omega }{\left({rx}+\displaystyle \frac{c}{t}\right)}^{2}.\end{eqnarray*}

Hence, at a fixed position *x*, the wavelength decreases over time, even though the source emits waves with a constant frequency *ω*. The phase pattern becomes stationary for large times because $\lambda (x,t)$ becomes time-independent in the large-time limit. The frequency ${\rm{\Omega }}=\partial \phi /\partial t$ seen by an observer at rest with position *x* is given by

32 \begin{eqnarray*}{\rm{\Omega }}=\displaystyle \frac{\omega }{{(1+{rxt}/c)}^{2}},\end{eqnarray*}

which depends on position and time. For $x\gt 0$ and $t\gt 0,$ ${\rm{\Omega }}=\omega +{{\rm{\Omega }}}_{{\rm{W}}}$, where ${{\rm{\Omega }}}_{{\rm{W}}}\lt 0.$ The wave pattern described by equation (30) is shown as a kymograph in figure 8(B). This pattern can be used to illustrate the dynamic wavelength effect by considering two observers at rest with different positions. An observer at rest that is more distant from the source (solid white line) crosses a smaller number of wave peaks compared to an observer closer to the source (dashed white line). Hence, the observer more distant from the source observes a smaller frequency.

Doppler effects are commonly found in wave physics. However, the dynamic wavelength effect is more unconventional. A time-dependent index of refraction as illustrated here occurs, e.g., in gases ionized by laser pulses due to a spatially and temporally inhomogeneous distribution of free electrons [23, 24].

## 6. Discussion

In this paper, we have introduced a continuum model of coupled phase oscillators in a dynamic medium to capture the dynamics of vertebrate segmentation. For simplicity, we have considered frequency and velocity profiles that scale with the time-dependent PSM length. Note that the phase profile itself does not scale in contrast to an earlier proposal [25]. Extending previous work [3, 13, 15, 19, 26], our approach takes into account tissue deformation due to growth of the embryonic body axis and the change of the PSM length over time. This enables us to quantitatively account for the morphological features of segmentation such as the timing of segment formation and the length of newly formed segments as observed in developing zebrafish embryos. The frequency and velocity profiles that scale with PSM length capture well the time-dependence of the experimentally observed wave patterns. The parameters obtained from the fit to the experimental data suggest that the frequency profile at the anterior end jumps from a finite value to zero. Such a behavior could, e.g., be caused by a Hopf bifurcation. Indeed, if the cellular oscillations pass a Hopf bifurcation from the oscillating state to the non-oscillating state when reaching the anterior end of the PSM, this would give rise to a frequency jump. Moreover, our theory describes the experimentally observed Doppler and dynamic wavelength effects, which regulate the timing of segment formation [4]. In particular, our results imply that the rate of segmentation in zebrafish is faster than the fastest local oscillation frequency found anywhere in the system. This remarkable behavior is due to the interplay of wave patterns and tissue shortening. The Doppler and dynamic wavelength effects observed in zebrafish are a result of the shortening of the PSM and the corresponding decrease in the local wavelength of the wave pattern. We predict these effects in general to occur also in other species. However, the signs and their role during different developmental stages could vary. The signaling pathways and the cellular processes that regulate and mediate the shortening of the PSM, the elongation of the body axis, and the specification of the frequency profile are as yet unknown and remain open challenges for future experimental and theoretical research.

## Figures and Tables

**Figure A1. njp519155f9:**
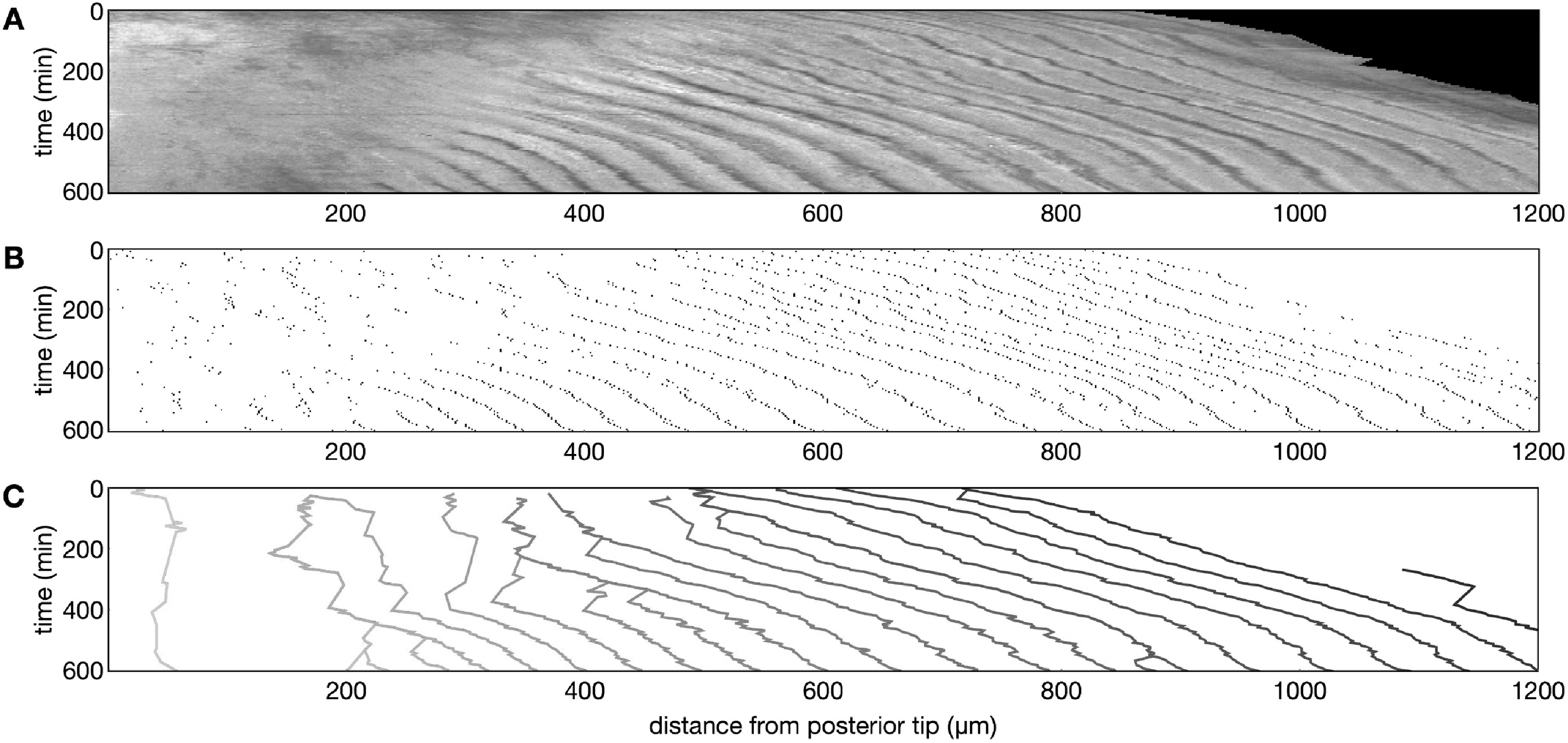
(A) Intensity kymograph obtained from a time-lapse microscopy movie of zebrafish segmentation. The *x*-axis marks the distance along the coordinate system defined in figure 1(D). (B) Algorithmically determined point set of local intensity minima indicating the position of segment boundaries. (C) Sets of adjacent points were algorithmically grouped to obtain separate traces for each segment boundary (different colors indicate different boundaries).

**Figure A2. njp519155f10:**
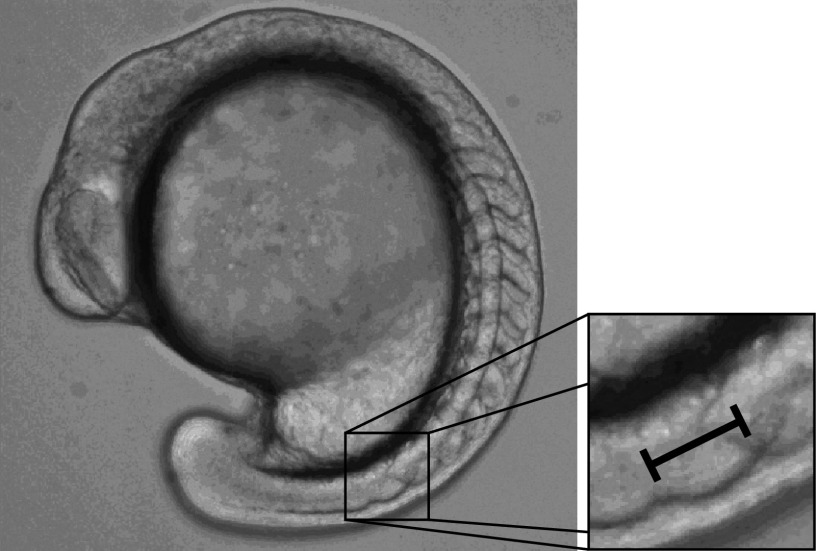
Measurement of the segment length at time of formation from brightfield microscopy time-lapse movies. Quantification procedure adapted from [21].

## Appendix A Quantification of segmentation dynamics from time-lapse microscopy movies

We use the time-dependent phase profiles of the transgenic zebrafish reporter line *Looping* determined previously [4]. In embryos of the same transgenic line, we quantified the cell velocity field in the segmented region and the length of segments at the time of formation from brightfield movies. These embryos developed at a temperature of 23.5 °C. For each frame of the movie, we defined a curved coordinate axis according to figure 2(A). We obtain the intensity values along this axis for each frame using FIJI image analysis software [27]. This procedure yields the kymograph figure A1 [Fig njp519155f9](A), which shows the profile of intensity values along the body axis as a function of time. In figure A1(A), dark gray lines indicate the segment boundaries. The slopes of these lines correspond to the speed of the segment boundaries relative to the posterior tip and thus carry information about the velocity field in the segmented region. To track the motion of the segment boundaries systematically, a peak-finding and tracking algorithm for the intensity level was developed and applied to a contrast-enhanced version of figure A1(A) smoothened with a moving average of width 12 pixels. Subsequently, the local intensity minima, which correspond to the positions of the segment boundaries, are determined with a peak-finding algorithm. The result is shown in figure A1(B). In the next step, nearby points are connected to obtain time series of the segment boundaries’ positions. The resulting traces are shown in figure A1(C). For each segment boundary, we perform linear fits of the boundary position at early and late times to determine its velocity. To obtain a velocity profile, we compute the average position of each segment boundary and assign the velocity of the corresponding boundary to it (figure 4(B)). The velocity profile within the PSM is inaccessible with the available dataset.

The segment length at the time of segment formation (figure 6(D)) was determined from these time-lapse microscopy movies following the procedure described in [21]. Specifically, the segment length was obtained by determining the distance between two successive indentations of the PSM at the anterior end of the tissue (figure A2 [Fig njp519155f10]).

## Appendix B Fits of theoretical phase profiles to experimental data

We use the shape parameters *σ*, *k*, and *q* of the frequency and velocity profiles as fit parameters. To generate dynamic patterns in our theory, we start the system at time ${t}_{0}\lt 0$ with $\phi (x,{t}_{0})=0$ to create initial conditions at *t* = 0. We fit the calculated patterns $\phi (x,t)$ for $t\geqslant 0$ to the experimental data, using *σ*, *k*, *q*, and *t*_0_ as fit parameters. The time *t* = 0 corresponds to the formation time of the 7th segment. The experimental phase map is obtained as an average over 18 embryos as described in [4]. Fits are performed by minimizing the squared average difference

B.1 \begin{eqnarray*}R={\displaystyle \int }_{0}^{{t}_{\mathrm{final}}}{\rm{d}}t{\displaystyle \int }_{0}^{\bar{x}(t)}{\rm{d}}x\;{(\psi (x,t)-{\psi }_{\mathrm{exp}}(x,t))}^{2}\end{eqnarray*}

between the theoretical and the experimental time-dependent phase profiles *ψ* and ${\psi }_{\mathrm{exp}}.$ Here, $\bar{x}(t)$ is the time-dependent PSM length, equation (14). We minimize equation (B.1) using a stochastic optimization process. Starting from an initial parameter choice, a random parameter set is created by adding numbers from Gaussian distributions with zero mean and specified variance to the reference parameter set. We only consider parameter sets for which the initial number of waves matches the observed one within 10% of the standard deviation (first data point in figure 7(B)). If such a random parameter set leads to a reduction of *R*, equation (B.1), it is chosen as a new reference parameter set. The algorithm converges to an optimal parameter region which can be refined by reducing the variances of the Gaussian distribution.

## Appendix C Tissue shortening at constant velocity

We determine a relationship between the anterior and posterior frequencies in the limit of small coupling strength *ϵ* and constant velocity profile $v(x)={v}_{0}.$ Hence, we simplify equation (1) by

C.1 \begin{eqnarray*}\displaystyle \frac{\partial \phi }{\partial t}(x,t)+{v}_{0}\displaystyle \frac{\partial \phi }{\partial x}(x,t)={\omega }_{0}U(x/\bar{x}(t)),\end{eqnarray*}

where $\bar{x}(t)$ is the time-dependent PSM length and $U$ is the profile function given by equation (4). The general solution to this equation is given by

C.2 \begin{eqnarray*}\phi (x,t)=\varphi (t-x/{v}_{0})+\displaystyle \frac{{\omega }_{0}}{{v}_{0}}{\displaystyle \int }_{0}^{x}U\left(\displaystyle \frac{x^{\prime} }{\bar{x}(t-\displaystyle \frac{x-x^{\prime} }{{v}_{0}})}\right)\;{\rm{d}}x^{\prime} ,\end{eqnarray*}

where the function $\varphi (u)$ can be determined as follows. The partial derivatives of this solution are given by

C.3 \begin{eqnarray*}\displaystyle \frac{\partial \phi }{\partial t}(x,t)=\dot{\varphi }(t-x/{v}_{0})-{\omega }_{0}{\rm{\Lambda }}(x,t),\end{eqnarray*}

C.4 \begin{eqnarray*}{v}_{0}\displaystyle \frac{\partial \phi }{\partial x}(x,t)=-\dot{\varphi }(t-x/{v}_{0})+{\omega }_{0}{\rm{\Lambda }}(x,t)+{\omega }_{0}U(x/\bar{x}(t)),\end{eqnarray*}

where $\dot{\varphi }={\rm{d}}\varphi /{\rm{d}}u,$ and

C.5 \begin{eqnarray*}{\rm{\Lambda }}(x,t)=\displaystyle \frac{1}{{v}_{0}}{\displaystyle \int }_{0}^{x}x^{\prime} \displaystyle \frac{\dot{\bar{x}}(t-\displaystyle \frac{x-x^{\prime} }{{v}_{0}})}{\bar{x}{(t-\displaystyle \frac{x-x^{\prime} }{{v}_{0}})}^{2}}U^{\prime} \left(\displaystyle \frac{x^{\prime} }{\bar{x}(t-\displaystyle \frac{x-x^{\prime} }{{v}_{0}})}\right){\rm{d}}x^{\prime} ,\end{eqnarray*}

with $\dot{\bar{x}}={\rm{d}}\bar{x}/{\rm{d}}t$ and ${U}^{\prime }={\rm{d}}U/{\rm{d}}\xi .$ The explicit form of $\varphi (u)$ can now be found using initial and boundary conditions. We evaluate equation (C.4) at *x* = 0 using open boundary conditions, $(\partial \phi /\partial x){| }_{x=0}=0$ to obtain

C.6 \begin{eqnarray*}\dot{\varphi }(u)={\omega }_{0}.\end{eqnarray*}

for $u\gt 0.$ Using the initial condition $\phi {| }_{t=0}=0$ and evaluating equation (C.2) at *t* = 0, we can likewise find the solution for *φ* for $u\lt 0.$ According to equation (C.2), $u\gt 0$ describes the solution at positions $x\lt {v}_{0}t.$

We are interested in the behavior of the anterior frequency at large times, for which $\bar{x}(t)\lt {v}_{0}t,$ and thus only consider $\varphi (u)$ for $u\gt 0.$ Using equation (C.6) in equations (C.3) and (C.4), we find the anterior frequency ${{\rm{\Omega }}}_{{\rm{A}}}=({\rm{d}}/{\rm{d}}t)\phi (\bar{x}(t),t)$ as

C.7 \begin{eqnarray*}{{\rm{\Omega }}}_{{\rm{A}}}=\left[\left(1-\displaystyle \frac{\dot{\bar{x}}}{{v}_{0}}\right)\left(1-{\rm{\Lambda }}(\bar{x}(t),t)\right)+\displaystyle \frac{\dot{\bar{x}}}{{v}_{0}}U(1)\right]{\omega }_{0}\end{eqnarray*}

This expression holds for arbitrary time-dependence $\bar{x}(t).$ We now consider linear tissue shortening at constant velocity $\dot{\bar{x}}=-\bar{v}.$ Using this relation in equation (C.5), we find

C.8 \begin{eqnarray*}{\rm{\Lambda }}(\bar{x}(t),t)={-{\displaystyle \int }_{0}^{\bar{x}(t)}}_{}^{}\displaystyle \frac{\beta x}{{[(1+\beta )\bar{x}(t)-\beta x]}^{2}}U^{\prime} \left(\displaystyle \frac{x}{(1+\beta )\bar{x}(t)-\beta x}\right)\;{\rm{d}}x,\end{eqnarray*}

where

C.9 \begin{eqnarray*}\beta =\bar{v}/{v}_{0}\end{eqnarray*}

and $\bar{x}(t)={\bar{x}}_{0}-\bar{v}t.$ Using the variable transform $\xi =x/[(1+\beta )\bar{x}(t)-\beta x]$ reveals that ${\rm{\Lambda }}(\bar{x}(t),t)={{\rm{\Lambda }}}_{0}$ with

C.10 \begin{eqnarray*}{{\rm{\Lambda }}}_{0} &amp; = &amp; {-\displaystyle \int }_{0}^{1}\displaystyle \frac{\beta \xi }{1+\beta \xi }{U}^{\prime }(\xi )\;{\rm{d}}\xi \\ &amp; = &amp; {\rm{\Delta }}-\displaystyle \frac{\beta }{1+\beta }U(1),\end{eqnarray*}

is time-independent. In the second line of equation (C.10), we have integrated by parts and introduced Δ given by equation (20). Using the result (C.10) and $\dot{\bar{x}}=-\bar{v}$ in equation (C.7), we finally obtain

C.11 \begin{eqnarray*}{{\rm{\Omega }}}_{{\rm{A}}}=(1+\beta )(1-{\rm{\Delta }}){\omega }_{0}.\end{eqnarray*}

The posterior frequency Ω_P_ =$(\partial \phi /\partial t){| }_{x=0}$ can be obtained using equations (C.3) and (C.6), which yields ${{\rm{\Omega }}}_{{\rm{P}}}={\omega }_{0}.$ Thus, we can interpret equation (C.11) as a relation between Ω_A_ and Ω_P_. This completes the derivation of equation (19).
